# Report of Superintendent of Indiana Hospital for Insane

**Published:** 1849

**Authors:** 


### ARTICLE III.

*Fourth Annual Report of the Commissioners and Superintendent of the Hospital for the Insane to the General Assembly of the State of Indiana.* Indianapolis: J. D. Defrees, State Printer. (From Dr. Patterson, Superintendent.)

This institution, since the date of the report before us, (Nov. 1st, 1848,) has gone into operation so far as to receive between forty and fifty patients. Every feature of its operations looks right, except the apparatus for heating and ventilation, which have not yet been put into successful operation as we are informed; but we have no doubt they will work well as soon as provisions are made for building fires large enough to produce the requisite amount of heat for warming, and consumption of air sufficient for ventilation.

As the management of such an institution devolves almost exclusively upon the resident officers, and especially upon the superintendent and physician, it is a matter of the greatest importance that they should be well chosen. In this respect we have the highest hopes for the success of this young institution.

R. J. Patterson, M. D., superintendent and physician to the institution, has had five years experience in the treatment of insanity in the largest and most successful institution in the west, (Ohio Lunatic Asylum,) and is eminently endowed with the qualities of head and heart to enable him to fulfil the duties of his responsible post with profit to the unfortunate sufferers from mental alienation, and credit to himself and the institution under his care.

John Nutt, M. D., assistant physician, was a member o the graduating class of Rush Medical College for 1848. He is intelligent, industrious, energetic, kind in disposition, and affable in manners ; so that he may be regarded as well suited for his situation.

Mrs. Laura Ann Elliott, matron, having filled the same important station in the Ohio Lunatic Asylum for several years, with great success and entire satisfaction, enters upon the office in this institution understanding the duties and appreciating the responsibilities of the place fully. She is a lady of a strong mind, refined and agreeable manners, steady and firm in her purposes, kind in her disposition, and devout in her christian character. That she will continue to be such a matron as the mother would be willing to trust with the care of her daughter, and the husband his wife when insane, we have not a doubt.

Such is the organization of this institution, and bright the prospect of its career of usefulness.

The large number of applications that cannot be received, show the necessity for completing other parts of the building at an early day. E.

